# Radiocarbon Reveals Modern Carbon Exchange With Topsoil Inorganic Carbon in Drylands

**DOI:** 10.1111/gcb.71095

**Published:** 2026-09-04

**Authors:** Hui Wang, Jianbei Huang, Fernando T. Maestre, Guang Zhao, Nan Lu, Cong Wang, Weiliang Chen, De Shorn E. Bramble, Marion Schrumpf, Michaela A. Dippold, Yangjian Zhang, Sönke Zaehle, Bojie Fu, Susan Trumbore

**Affiliations:** ^1^ Department of Biogeochemical Processes Max Planck Institute for Biogeochemistry Jena Germany; ^2^ Geo‐Biosphere Interactions, Department of Geosciences University of Tübingen Tübingen Germany; ^3^ State Key Laboratory of Regional and Urban Ecology, Research Center for Eco‐Environmental Sciences Chinese Academy of Sciences Beijing China; ^4^ National Observation and Research Station of Earth Critical Zone on the Loess Plateau in Shaanxi Xi'an China; ^5^ Shaanxi Yan'an Forest Ecosystem Observation and Research Station Beijing China; ^6^ Environmental Science and Engineering, Biological and Environmental Science and Engineering Division King Abdullah University of Science and Technology Thuwal Kingdom of Saudi Arabia; ^7^ Lhasa Plateau Ecosystem Research Station, Key Laboratory of Ecosystem Network Observation and Modeling, Institute of Geographic Sciences and Natural Resources Research Chinese Academy of Sciences Beijing China; ^8^ Fujian Academy of Forestry Fuzhou China; ^9^ Faculty of Land and Food Systems University of British Columbia Vancouver Canada; ^10^ College of Resources and Environment University of Chinese Academy of Sciences Beijing China; ^11^ Department of Biogeochemical Signals Max Planck Institute for Biogeochemistry Jena Germany

**Keywords:** aridity, carbon isotope, drylands, modern carbon cycling, pedogenic carbonate, radiocarbon, soil inorganic carbon, soil pH

## Abstract

Drylands store most of the global soil inorganic carbon (SIC), yet the extent to which this pool interacts with contemporary carbon (C) cycling remains poorly understood. To test whether SIC behaves primarily as an inert geological reservoir or instead bears a modern carbon imprint, we quantified SIC content and radiocarbon (∆^14^C) at 42 dryland sites spanning broad aridity gradients across Eurasia. We also evaluated the climatic, edaphic, and biotic factors associated with variation in ∆^14^C‐SIC. Across all sites, topsoil SIC (~0–10 cm) was strongly depleted in ^14^C but consistently enriched relative to ^14^C‐dead carbonates, indicating that it contains a measurable component derived from modern carbon inputs. In the Chinese drylands, ∆^14^C‐SIC declined with increasing aridity, consistent with weaker modern carbon exchange under drier conditions and a greater contribution of inherited or ^14^C‐depleted carbonate carbon. Soil pH and ∆^14^C of soil organic carbon were the strongest predictors of ∆^14^C‐SIC, suggesting that carbonate dissolution‐reprecipitation and the age of carbon entering soil CO_2_ play key roles in determining SIC origins. At a subset of nine Chinese sites, ∆^14^C‐SIC declined sharply with depth and approached ^14^C‐dead values in subsoils, indicating little influence of modern carbon in deeper carbonate pools. The presence of mixed ^14^C‐depleted and modern ^14^C signatures in SIC potentially complicates the use of SIC isotopic signatures as proxies of environmental conditions. Together, our results indicate that dryland topsoil SIC commonly carries a measurable modern carbon signature that is tightly linked to contemporary carbon cycling. This coupling weakens with increasing aridity and soil depth, suggesting that environmental change in drylands may reshape one of the planet's largest carbon pools not only through changes in SIC stocks, but also through shifts in carbonate radiocarbon signatures and sources.

## Graphical abstract

Radiocarbon measurements across drylands reveal that topsoil inorganic carbon carries a measurable modern carbon signature linked to soil pH and organic carbon pools. This modern imprint weakens with increasing aridity and soil depth, indicating reduced exchange with contemporary carbon sources under drier conditions and in deeper soils.

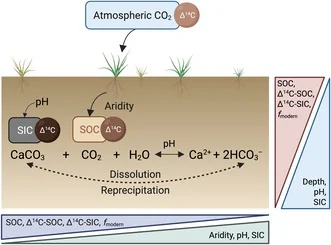

## 1 Introduction

Drylands, areas where the ratio of precipitation to potential evapotranspiration is lower than 0.65, cover more than 40% of the Earth's land surface (Lal 2019; Prăvălie 2016; Reynolds et al. 2007). However, these regions are experiencing accelerating land degradation and desertification under climate change (Huang et al. 2016; Lal 2009; Maestre et al. 2025). Dryland soils store approximately 95% of the global soil inorganic carbon (SIC) to a depth of 2 m (~2300 Pg C; Huang et al. 2024; Lal 2019), a stock generally larger than that of soil organic carbon (SOC), particularly in deeper soil layers (Huang et al. 2024; Plaza et al. 2018). Most studies of soil carbon cycling in drylands have focused on the dynamics of SOC (Díaz‐Martínez et al. 2024; Gaitán et al. 2019), while the role of SIC has been largely overlooked (Raza et al. 2024). This is partly because SIC has long been viewed as a geochemically stable pool with turnover times of millennia (Sanderman 2012; Zamanian et al. 2021). However, increasing evidence suggests that SIC content may respond to climate and land use change on decadal timescales (Song et al. 2022; Zeng et al. 2023; Zhang et al. 2025), indicating that SIC may play a more active role in contemporary carbon cycling than previously assumed. Determining whether SIC is predominantly an inert geological reservoir or a dynamic pool linked to contemporary carbon cycling is therefore crucial for assessing how SIC will respond to climate and land use change.

Most arid soils contain SIC either as geogenic carbonates derived from the weathering of calcareous parent materials or as pedogenic carbonates precipitated from soil pore‐space CO_2_ that reflect recently fixed carbon (Zamanian et al. 2016). Pedogenic carbonates may form either through recrystallization following dissolution of inherited calcareous material or as new carbonate minerals formed using Ca or Mg released during silicate weathering or from dust deposition (Mi et al. 2008; Zamanian et al. 2016). The mechanisms of dissolution and precipitation of pedogenic carbonates are as follows:

(1) $${\mathrm{CO}}_{2}+H_{2}O⇌{\mathrm{HCO}}_{3^{–}}+H^{+}$$

(2)

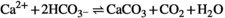




The carbonate precipitation–dissolution equilibrium is controlled by a combination of climatic, biological, and edaphic factors. In drylands, limited water inputs and high evaporation rates keep soil solutions concentrated in Ca^2+^ and bicarbonate (HCO_3_^−^), pushing the equilibrium toward carbonate precipitation rather than dissolution (Breecker et al. 2010), and at the same time reducing the downward transport of bicarbonates (Zhang et al. 2025). Elevated soil pH further shifts the equilibrium toward HCO_3_^−^ and carbonate ions (CO_3_^2−^), thereby enhancing carbonate precipitation and reducing carbonate dissolution (Zamanian et al. 2016). In addition, increasing plant cover (Pan et al. 2022) can reduce SIC stocks through plant uptake of soil base cations (Sartori et al. 2007) and lowering soil pH (Huang et al. 2022). The isotopic composition of pore‐space CO_2_ equilibrating with precipitating carbonates reflects a combination of atmospheric CO_2_ diffusing into soils, root respired CO_2_ and CO_2_ released from SOC decomposition.

Radiocarbon (^14^C) is a powerful tracer for distinguishing modern ^14^C signals from “geogenic” ^14^C‐depleted carbon and for assessing soil carbon dynamics across timescales (Trumbore 2009). Although radiocarbon has been widely used to investigate the age and turnover of SOC (Trumbore 2000), its application to SIC remains limited to paleoclimate research (Chen and Polach 1986; Dina Ebouel et al. 2024). Specifically, radiocarbon allows us to detect whether modern carbon, including “bomb ^14^C” released during atmospheric nuclear weapons testing in the early 1960s, has exchanged with pedogenic carbonates via precipitation–dissolution processes on decadal timescales (Trumbore 2009). In contrast, geogenic carbonates are dominated by lithogenic carbon, in which ^14^C decay over timescales longer than ~60,000 years has removed all ^14^C (Trumbore 2000, 2009). Thus, unlike SIC stock measurements alone, ^14^C can reveal whether carbonate carbon has remained isolated from modern carbon for tens of thousands of years or has exchanged with modern carbon on decadal timescales. Radiocarbon itself does not distinguish net formation of new pedogenic carbonates from isotopic exchange or recrystallization of ^14^C‐depleted carbonate minerals under conditions where they can equilibrate with modern or “bomb” CO_2_. Such insights are crucial for assessing the persistence of SIC (Huang et al. 2024) and for evaluating the extent to which *δ* ^13^C values of SIC can be used to infer past vegetation changes in paleosols (Rao et al. 2006).

Here, we quantified the ∆^14^C of SIC in dryland soils from 42 sites across China, Iran, and Spain to test whether SIC acts primarily as an inert geological reservoir or instead interacts with modern carbon cycling across broad environmental gradients. Specifically, we used radiocarbon measurements of SIC, SOC, and soil respired CO_2_ to assess the extent to which topsoil SIC bears an imprint of modern carbon and to evaluate how this imprint varies with climate, vegetation, and soil properties. We further examined soil profiles from a subset of nine Chinese dryland sites to determine how SIC radiocarbon signatures change with depth. We hypothesized that the modern carbon imprint on SIC would decline with increasing aridity and soil depth, reflecting reduced carbonate turnover and a greater contribution of inherited or strongly ^14^C‐depleted carbonate carbon under drier conditions and in subsoils. We further hypothesized that ∆^14^C‐SIC would be most strongly associated with soil pH and the radiocarbon signature of SOC because carbonate dissolution and reprecipitation and the age of carbon entering soil CO_2_ should together influence the presence of modern carbon in SIC. By focusing on SIC radiocarbon rather than stock size alone, our study aims to provide a more process‐based assessment of the persistence and environmental sensitivity of inorganic carbon in dryland soils.

## 2 Materials and Methods

### 2.1 Soil Sampling

Soil samples were collected from 42 sites across China, Iran, and Spain (Figure S1) between June 2016 and July 2020. The broad geographic distribution spans a wide range of aridity (calculated as 1 − aridity index: 0.46 to 0.99), mean annual temperature (MAT: −3.4°C to 17.4°C), and vegetation types, including forest, grassland, shrubland, desert, and alpine meadow. All sites were either ungrazed or only lightly grazed in the years prior to sampling.

At sites in Iran and Spain, topsoils (0–7.5 cm) were sampled as part of the BIODESERT global survey as described in Maestre et al. (2022). At all Chinese sites, soils were collected from the top 10 cm using the same BIODESERT sampling design. At nine Chinese sites, midsoil (40–50 cm) and subsoil (90–100 cm) were also sampled concurrently with topsoil. For each site, soil collected from five quadrats or plots was gently mixed to form a homogeneous composite sample and transported to the laboratory. In the laboratory, samples were then passed through a 2 mm sieve and air‐dried for subsequent analyses, including physicochemical characterization and incubation experiments.

### 2.2 Incubation and Radiocarbon Measurement

To determine the radiocarbon (^14^C) of respired CO_2_, 30 g of dry soil from each site was incubated in airtight flasks at 20°C after adding water until soils were at 60% of water holding capacity. Following water addition, the flasks were immediately sealed and flushed with synthetic air to remove CO_2_ from the headspace. An open tube containing 5 mL of Milli‐Q water was placed in each flask to maintain humidity during incubation. Respired CO_2_ concentrations were monitored with a gas analyzer (LI‐COR 6262, Lincoln, NE, USA). Once the cumulative carbon release exceeded 0.2 mg, headspace gas was collected for radiocarbon analysis (Wang et al. 2026).

For SIC radiocarbon measurements, a soil subsample equivalent to approximately 0.8 mg C was placed in a sealed vial and acidified with H_3_PO_4_ to release CO_2_ from inorganic carbon. CO_2_ derived from these pretreated samples, together with respired CO_2_ collected during incubation, was purified and subsequently graphitized with H_2_ at 600°C. The graphitized samples were analyzed using an accelerator mass spectrometer (AMS; Micadas, Ionplus, Switzerland) (Steinhof et al. 2017).

Radiocarbon values are reported as ∆^14^C, which expresses the deviation (‰) of the ^14^C/^12^C ratio from the modern standard after correction for radioactive decay since 1950 and for mass‐dependent fractionation (Trumbore 2000). ∆^14^C was calculated as:

(3) $${\text{∆}}^{14}C=\left(F^{14}C\times e^{\mathrm{\lambda c}\;\left(1950-t\right)}–1\right)\times 1000$$

where *F* ^14^C is the fraction modern, defined as the measured sample ^14^C/^12^C ratio divided by 0.95 times the measured ^14^C/^12^C ratio of the Oxalic Acid I standard (OX‐I), *λ*_*C*_ is the updated radiocarbon decay constant (1/8267 year^−1^), and *t* is the year of soil sampling. To account for differences in mass‐dependent fractionation, *F* ^14^C was normalized using the difference between the measured *δ* ^13^C of each sample and a common *δ* ^13^C value of −25‰, assuming that ^14^C is fractionated twice as strongly as ^13^C (Mook and van der Plicht 1999).

Conventional radiocarbon age (*τ*, years) was calculated from *F* ^14^C as follows:

(4) $$\tau =-8033\times \ln\;\left(F^{14}C\right)$$

where 8033 years is the mean life of radiocarbon derived from the Libby half‐life of 5568 years (Mook and van der Plicht 1999).

### 2.3 The ^14^C‐Derived Modern Carbon Fraction of SIC

To estimate the relative proportions of modern and ^14^C‐depleted carbon within SIC, we calculated a ^14^C‐derived apparent modern carbon fraction in SIC (*f*_modern_) using the following mass balance Equation (5):

(5) $${\text{∆}}^{14}C‐\mathrm{SIC}=f_{\text{modern}}\times {\text{∆}}^{14}C‐{\mathrm{SIC}}_{\text{modern}}+\left(1–f_{\text{modern}}\right)\times {\text{∆}}^{14}C‐{\mathrm{SIC}}_{\text{depleted}}$$

where ∆^14^C‐SIC is the measured radiocarbon value of the isolated SIC fraction, ∆^14^C‐SIC_modern_ represents the radiocarbon signature of carbonate carbon influenced by modern carbon inputs (i.e., carbon that has equilibrated with soil pore‐space CO_2_), and ∆^14^C‐SIC_depleted_ refers to the radiocarbon signature of stabilized carbonate pools. ∆^14^C‐SIC_depleted_ is assumed to have ∆^14^C of −1000‰ (*F* ^14^C = 0), and can include inherited geogenic carbonates as well as ancient pedogenic carbonates (> 60,000 years old) that have not exchanged carbon with modern sources.

We used two assumptions to span the potential range of ∆^14^C‐SIC_modern_ to reflect the fact that the soil pore‐space CO_2_ can have a range of ∆^14^C values. First, we assumed that the ∆^14^C of CO_2_ in pore space is the same as that of CO_2_ respired during incubations. This represents the end‐member case in which pore‐space CO_2_ is dominated by local microbial decomposition of soil organic matter. Under field conditions, soil pore‐space CO_2_ will also include more modern components, including atmospheric CO_2_, root respiration, and decomposition of recent plant residues (Zamanian et al. 2016), which were removed before our incubations. The second assumption (reported as *f*_modern_′ in Table S1) used the atmospheric ∆^14^C signature in the sampling year as ∆^14^C‐SIC_modern_′. This is equivalent to assuming that pore‐space CO_2_ is dominated by autotrophic respiration. Although these two assumptions represent contrasting heterotrophic‐ and autotrophic‐dominated scenarios, the resulting *f*_modern_ and *f*_modern_′ values were similar (Table S1). This is because both assumed modern sources were much younger than the nearly ^14^C‐free carbonate carbon.

We emphasize that *f*_modern_ and *f*_modern_′ should be interpreted as estimates of the apparent modern carbon fraction of SIC rather than as direct estimates of newly precipitated carbonate. Radiocarbon results cannot by themselves distinguish between SIC that has dissolved, equilibrated with pore‐space CO_2_ and reprecipitated in situ (i.e., with no net storage or release of carbon), and the accumulation of new pedogenic carbon in a horizon dominated by geogenic SIC. Differentiating these two cases would require additional information about changes in SIC over time or isotopic information allowing new sources of Ca to be distinguished from those of geogenic origin.

### 2.4 Stable Isotope Analysis

The stable isotope signature (*δ* ^13^C) of total soil carbon (TC) and SOC was determined using an elemental analyzer (Carlo Erba 1100 ce analyzer; Thermo Fisher Scientific) coupled to an isotope ratio mass spectrometer (IRMS; Delta+ XL; Thermo Fisher Scientific) via a ConFlow III open‐split system (Finnigan MAT, Bremen, Germany) (Lange et al. 2023). The *δ* ^13^C of SIC was then calculated by the following mass balance Equation (6):

(6) $$\delta^{13}{\text{C}}_{\text{SIC}}=\frac{\delta^{13}C_{\mathrm{TC}}\times w_{\mathrm{TC}}–\delta^{13}{\text{C}}_{\text{SOC}}\times w_{\mathrm{SOC}}}{w_{\mathrm{SIC}}}$$

where *δ* ^13^C_TC_, *δ* ^13^C_SOC_, and *δ* ^13^C_SIC_ are the *δ* ^13^C values of TC, SOC, and SIC, respectively, and *w*_TC_, *w*_SOC_, and *w*_SIC_ are the corresponding carbon contents. Additional *δ* ^13^C‐based calculations used to derive *δ* ^13^C‐CO_2_‐pore, *δ* ^13^C‐CO_2_‐equilibrium, *δ* ^13^C‐CO_2_‐plant, and the ^13^C‐derived modern carbon fraction in SIC (*F*_modern_) are provided in Supplementary Text and Table S4.

### 2.5 Climate, Vegetation and Soil Variables

Mean annual temperature (MAT) was obtained from WorldClim for the 1970–2000 period (Fick and Hijmans 2017). Aridity index was taken from the Global Aridity Index database (Zomer et al. 2022); for clarity, aridity was expressed as 1 – aridity index (AI), with higher values indicating drier conditions (Delgado‐Baquerizo et al. 2013). Net primary productivity (NPP) was downloaded from MODIS Collection 6.1 (2000–2020) at 500 × 500 m spatial resolution (Running and Zhao 2021). Because our analyses addressed broad regional differences among sites, we used MODIS NPP to characterize the long‐term productivity setting around each sampling location. Despite its coarser resolution relative to plot size, MODIS provides a consistent and comparable productivity estimate across all sites, which is appropriate for evaluating large‐scale climatic and biotic controls in this distributed dryland survey. Soil pH was measured in a 1:2.5 soil‐to‐water suspension using a glass pH electrode (pH 538, WTW, Weilheim, Germany). Soil texture (clay, silt, and sand fractions) was determined by the pipette method (Kettler et al. 2001). Cation exchange capacity (CEC) was determined by ammonium acetate extraction at pH 7, and the concentrations of exchangeable cations were measured by inductively coupled plasma−optical emission spectrometry (ICP − OES, Ultima 2, Horiba Jobin‐Yvon, Longjumeau, France) (Hendershot et al. 2008). SIC was quantified by heating samples at 450°C to remove organic carbon, followed by dry combustion using a vario MAX Cube elemental analyzer (Elementar Analysensysteme GmbH, Langenselbold, Germany) (Bramble et al. 2024). SOC content was then calculated as the difference between TC (determined by dry combustion at 1100°C) and SIC.

### 2.6 Statistical Analysis

We used a multiple linear regression model to compare the relative effect sizes of potential environmental drivers (MAT, aridity, NPP, soil pH, soil clay and silt content, CEC, SIC, SOC, ∆^14^C‐SOC, and ∆^14^C‐CO_2_) on topsoil ∆^14^C‐SIC (Table S2). Because replication within groups was limited and unbalanced, mixed‐effects models were not robustly supported by the data. We therefore included region as a fixed categorical predictor to compare standardized effect sizes among environmental drivers. Before modeling, NPP was natural‐log transformed to reduce skewness. All numeric predictors were then z‐standardized (mean = 0, standard deviation = 1) to place predictors on the same scale (Schielzeth 2010). After fitting, we evaluated model assumptions using residual diagnostic tests. Residual normality was assessed using a one‐sample Kolmogorov–Smirnov test (Lilliefors 1967), homoscedasticity using the Breusch–Pagan test (Breusch and Pagan 1979), and multicollinearity using adjusted variance inflation factors (VIFs) (Kim 2019).

After identifying the main correlates of topsoil ∆^14^C‐SIC across environmental drivers using multiple regression, we then used a piecewise structural equation model (piecewise SEM) to evaluate a hypothesized pathway linking aridity to ∆^14^C‐SIC in the Chinese regions, where aridity relationships were most evident. Aridity was treated as the focal exogenous driver, and pH, ∆^14^C‐SOC, SIC, and SOC were included based on the multiple regression results and their mechanistic relevance. The hypothesized model comprised five component linear models: (1) pH~aridity, (2) SOC~aridity, (3) ∆^14^C‐SOC~aridity + pH, (4) ∆^14^C‐SIC~aridity + pH + ∆^14^C‐SOC, and (5) SIC~aridity + pH + ∆^14^C‐SIC, with correlated residuals specified between pH and SOC and between SOC and ∆^14^C‐SOC. We additionally evaluated alternative candidate models incorporating NPP, clay + silt, or ∆^14^C‐CO_2_, and compared all models using Fisher's C statistic and AIC. The model including aridity, pH, SOC, SIC, ∆^14^C‐SOC, and ∆^14^C‐SIC had the lowest AIC (592.531) and showed acceptable overall fit (Fisher's C = 9.288, *p* = 0.158), and was therefore retained as the final model, whereas models including NPP, clay + silt, or ∆^14^C‐CO_2_ showed either poorer fit or higher AIC values (Table S3). Standardized path coefficients and their significance were extracted from the retained model, and *R* ^2^ values were obtained for each response variable. Indirect effects of aridity on ∆^14^C‐SIC were estimated by multiplying standardized coefficients along each indirect pathway.

A sensitivity analysis was performed to evaluate the influence of analytical uncertainty on *f*_modern_. For each sample, a central *f*_modern_ value was first calculated from the measured ∆^14^C‐SIC and ∆^14^C‐SIC_modern_ values. To estimate the sensitivity range, ∆^14^C‐SIC was shifted downward and upward by its analytical error, while ∆^14^C‐SIC_modern_ was shifted in the opposite direction to obtain the minimum and maximum plausible *f*_modern_ values. For each soil layer, the average *f*_modern_ was calculated as the arithmetic mean of the nine sample central values, and the corresponding sensitivity ranges were reported as the minimum to maximum intervals (Table S1). The same sensitivity analysis method was used for *f*_modern_′ (Table S1).

To compare the ∆^14^C‐SIC, *f*_modern_, SIC content, ∆^14^C‐CO_2_, *δ* ^13^C‐SIC, and pH in soil layers, we treated soil profile as a paired factor because topsoil, midsoil, and subsoil measurements were obtained from the same profile. Differences among the three soil depths were assessed using Friedman tests. When the overall depth effect was significant, post hoc pairwise comparisons were performed using Wilcoxon signed‐rank tests with Holm adjustment for multiple comparisons.

All analyses were performed in R version 4.4.1 using the *stats*, *car*, and *piecewiseSEM* packages (Lefcheck 2016).

## 3 Results

**FIGURE 1:**
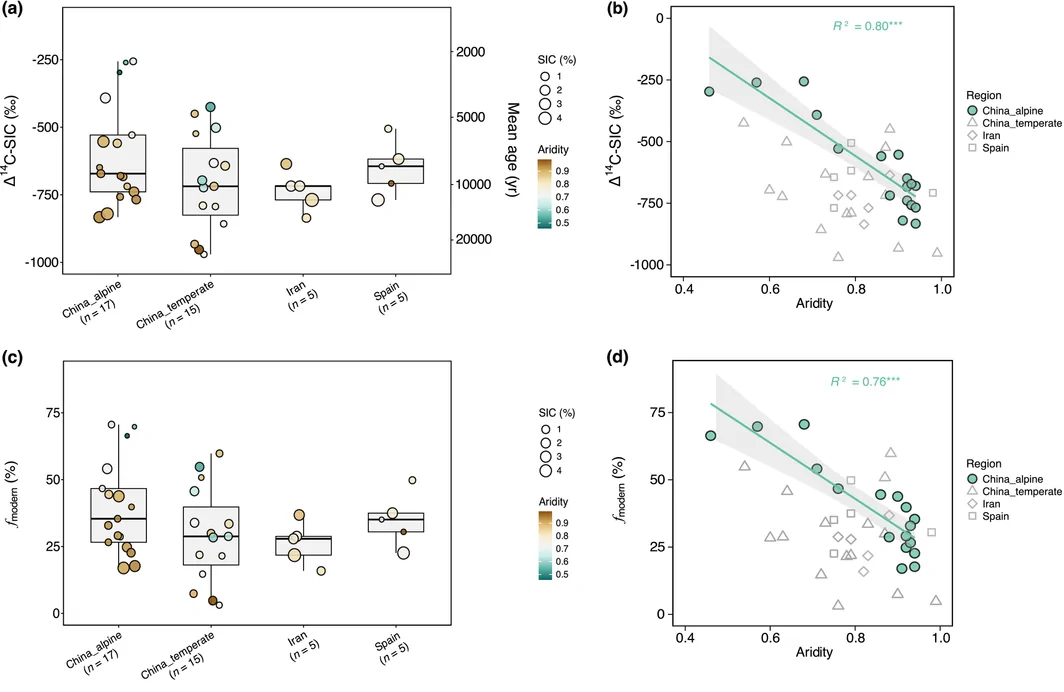
Distribution of ∆^14^C‐SIC and the radiocarbon‐derived modern carbon fraction of SIC (*f*_modern_) in dryland topsoils. (a, c) Regional distributions of ∆^14^C‐SIC (a) and *f*_modern_ (c) across four dryland regions. Points are colored by aridity and scaled by SIC content (%). Boxplots show the median and interquartile range, with whiskers representing the data spread. In panel (a), the right *y*‐axis indicates the corresponding mean conventional ^14^C age of SIC (year), calculated using the Libby half‐life. (b, d) Relationships of ∆^14^C‐SIC with aridity (b) and of *f*_modern_ with aridity (d) across the four regions. Numbers in the panels show the sample size (*n*) for each region. For the China_alpine samples, the green solid line shows the fitted linear regression, with gray shading indicating the 95% confidence interval (CI). The coefficients of determination (*R* ^2^) for each fitted relationship are shown in panels (b) and (d). Significance levels are indicated as ***p < 0.001. China_alpine, Qinghai–Tibetan Plateau in China; China_temperate, temperate Inner Mongolia and the Loess Plateau in China. Δ^1^ ^4^C‐SIC, Δ^14^C of soil inorganic carbon.

Across topsoils from four different Eurasian dryland regions, ∆^14^C‐SIC values were all below −250‰, and the modern carbon fraction in SIC (*f*_modern_) ranged from nearly 0 to about 75% (Figure 1a,c). The relationships with aridity were most evident in the Chinese drylands. In Chinese alpine drylands, ∆^14^C‐SIC declined significantly with increasing aridity (Figure 1b). Accordingly, *f*_modern_ also showed a corresponding decline (Figure 1d). In Chinese temperate drylands, the relationships with aridity were weak when all samples were included (∆^14^C‐SIC: *R* ^2^ = 0.14; *f*_modern_: *R* ^2^ = 0.27). However, after excluding two high‐leverage sites (ALSHS and SNTY1) with high aridity but unusually low soil pH and coarse texture, both relationships became stronger and statistically significant (∆^14^C‐SIC: *R* ^2^ = 0.46, *p* < 0.05; *f*_modern_: *R* ^2^ = 0.46, *p* < 0.01; Figure S2). Compared with the Chinese dryland datasets, the Iran and Spain datasets included fewer sampling sites and spanned narrower gradients of aridity (Iran, 0.76–0.88; Spain, 0.75–0.98), soil pH (Iran, 7.72–7.88; Spain, 6.44–7.57), and Δ^14^C‐SIC (Figure 1), thereby reducing the statistical power to detect significant relationships.

**FIGURE 2:**
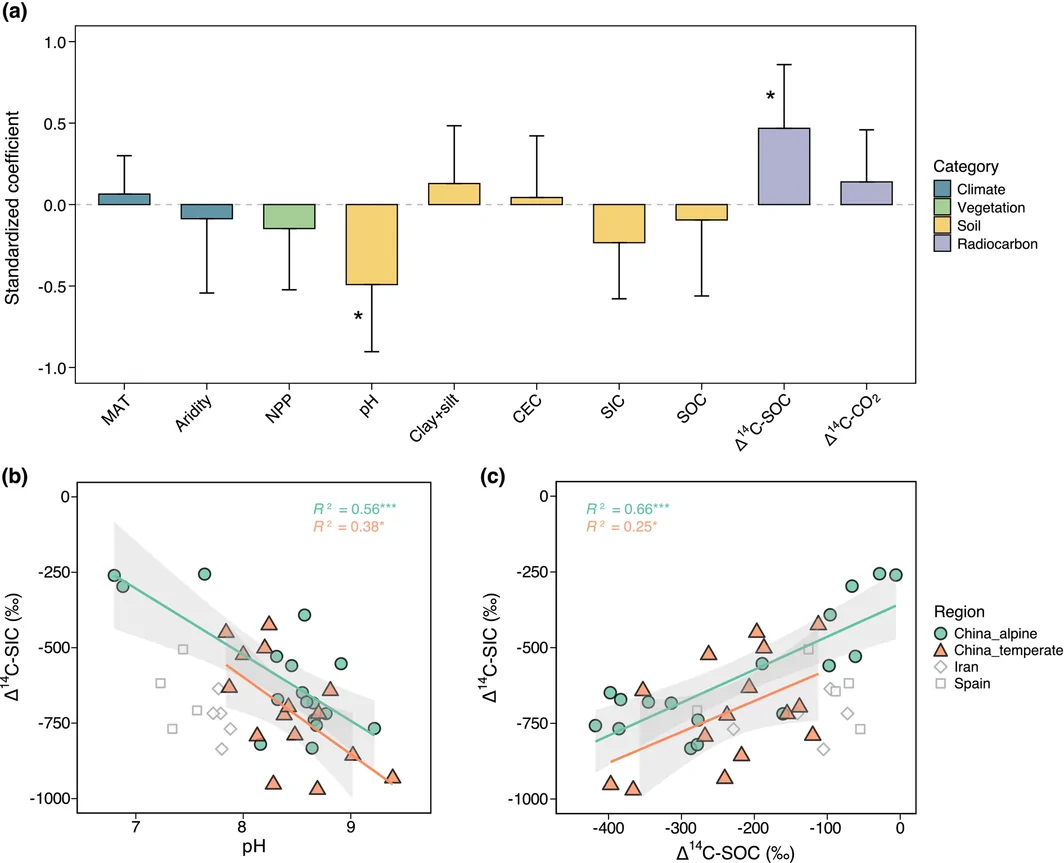
Effects of environmental drivers on ∆^14^C of SIC in dryland topsoils. (a) Effects of environmental predictors on ∆^14^C‐SIC estimated by multiple linear regression using all 42 topsoil samples. Colored bars denote standardized regression coefficients and black error bars indicate 95% confidence intervals (CIs). Predictors are grouped into four categories: climate, vegetation, soil, and radiocarbon‐related variables. The horizontal dashed line indicates a coefficient of zero. The model explained 66% of the variance in ∆^14^C‐SIC (*n* = 42). Asterisks indicate significant predictors (*p < 0.05). (b, c) Relationships between ∆^14^C‐SIC and pH (b), and between ∆^14^C‐SIC and ∆^14^C‐SOC (c) across four regions. Symbols represent individual samples from China_alpine, China_temperate, Iran, and Spain. The green and orange lines indicate fitted linear regressions for China_alpine and China_temperate, respectively, and the gray shading denotes the 95% CI of each regression. Significant linear relationships were observed for samples from the Qinghai–Tibetan Plateau and from temperate Inner Mongolia and the Loess Plateau in China. The coefficients of determination (*R* ^2^) are shown in panels (b) and (c). Significance levels are indicated as *p < 0.05, and ***p < 0.001. China_alpine, Qinghai–Tibetan Plateau in China; China_temperate, temperate Inner Mongolia and the Loess Plateau in China. MAT, mean annual temperature; NPP, net primary productivity; CEC, cation exchange capacity; SIC, soil inorganic carbon; SOC, soil organic carbon; ∆^14^C‐SOC, ∆^14^C of SOC; ∆^14^C‐CO_2_, ∆^14^C of respired CO_2_; ∆^14^C‐SIC, ∆^14^C of SIC.

To identify the main controls on ∆^14^C‐SIC, we fitted a multiple linear regression model to all 42 topsoil samples, including climatic factors, NPP, soil properties, and ∆^14^C of SOC and respired CO_2_. The model explained 66% of the variation in ∆^14^C‐SIC (*R* ^2^ = 66%, adjusted *R* ^2^ = 51%, *F*_13,28_ = 4.24, *p* < 0.001, *n* = 42; Figure 2a; Table S2). Among all predictors, soil pH was negatively associated with ∆^14^C‐SIC (estimate = −0.49, 95% CI = −0.92 to −0.06), whereas ∆^14^C‐SOC was positively associated with ∆^14^C‐SIC (estimate = 0.47, 95% CI = 0.06 to 0.88). Model diagnostics supported the overall suitability of the linear regression model, with approximately normal residuals (Kolmogorov–Smirnov test: *D* = 0.10, *p* = 0.80), no evidence of heteroscedasticity (Breusch–Pagan test: *χ* ^2^ = 10.94, *p* = 0.62), and low multicollinearity (maximum adjusted VIF = 2.17). Consistent with these model results, ∆^14^C‐SIC decreased with increasing pH in both Chinese alpine and temperate drylands (Figure 2b). Likewise, ∆^14^C‐SIC increased with ∆^14^C‐SOC in both regions (Figure 2c). Together, these analyses indicate that soil pH and ∆^14^C‐SOC are more important predictors of ∆^14^C‐SIC than climate or vegetation.

**FIGURE 3:**
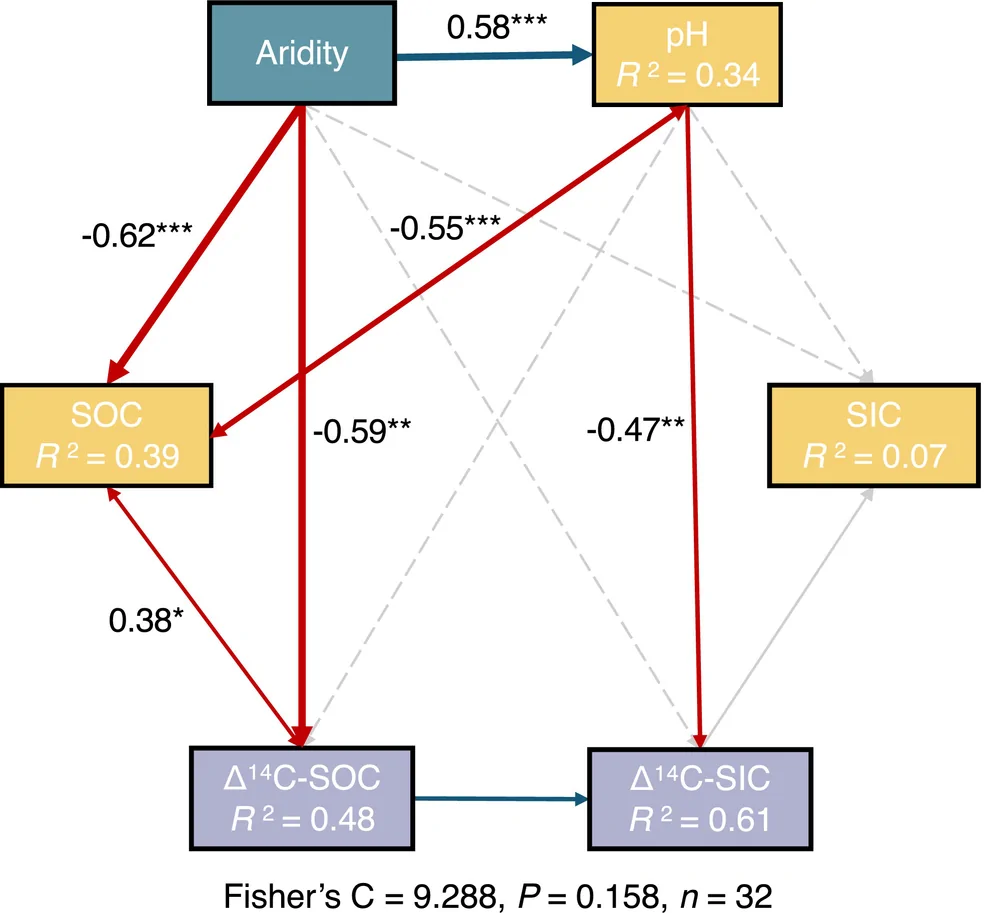
Piecewise structural equation model (SEM) showing the effects of aridity and soil properties on ∆^14^C‐SIC at Chinese sites. The SEM was restricted to the Chinese sites (China_alpine and China_temperate; *n* = 32) because Iran and Spain had limited samples and strong regional contrasts. Single‐headed arrows represent directional effects (regression paths), and double‐headed arrows represent covariances between variables. Blue and red solid arrows indicate significant positive and negative paths (*p* ≤ 0.05), respectively, while gray dashed arrows indicate nonsignificant paths retained in the model. Values associated with arrows are standardized path coefficients, and arrow width is proportional to the strength of the path coefficients. *R* ^2^ values shown within response variables indicate the proportion of variance explained by the model. Model fit statistics are shown below the panel (Fisher's C = 9.288, *p* = 0.158, *n* = 32). Significance levels are indicated as *p < 0.05, **p < 0.01, and ***p < 0.001. SOC, soil organic carbon; SIC, soil inorganic carbon; ∆^14^C‐SOC, ∆^14^C of SOC; ∆^14^C‐SIC, ∆^14^C of SIC.

The piecewise SEM was fitted only to the Chinese sites because Iran and Spain had a limited number of sites and a narrow range of aridity. For the selected sites, it explained 61% of the variance in ∆^14^C‐SIC (Figure 3). Aridity was associated with ∆^14^C‐SIC primarily through two indirect pathways. One pathway was mediated by soil pH: aridity was positively associated with pH (0.58, *p* < 0.001), and higher pH was associated with lower ∆^14^C‐SIC (−0.47, *p* < 0.01). The other pathway involved SOC and its ^14^C signature: aridity was negatively associated with both SOC (−0.62, *p* < 0.001) and ∆^14^C‐SOC (−0.59, *p* < 0.01), and lower ∆^14^C‐SOC values were associated with lower ∆^14^C‐SIC (0.45, *p* < 0.05). Together, these pathways are consistent with the interpretation that increasing aridity is linked to lower ∆^14^C‐SIC through changes in soil pH and reduced modern carbon inputs to soil organic matter.

**FIGURE 4:**
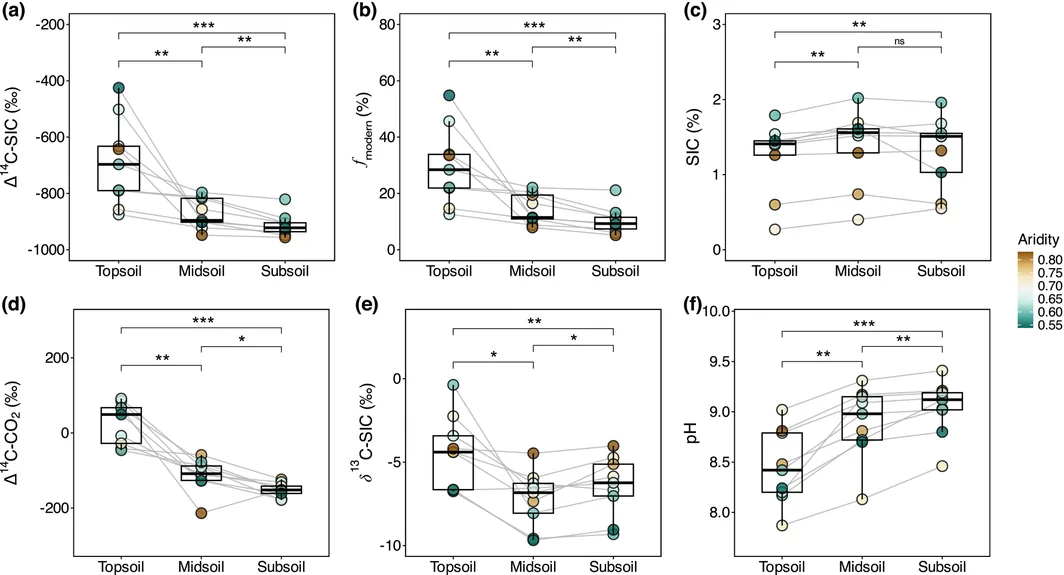
Variation in SIC isotopic signatures and related factors across soil layers. (a) ∆^14^C of SIC, (b) *f*_modern_, (c) SIC content, (d) ∆^14^C of respired CO_2_, (e) *δ* ^13^C of SIC, and (f) soil pH among topsoil (0–10 cm), midsoil (40–50 cm), and subsoil (90–100 cm). Boxplots show the median and interquartile range, with whiskers indicating the data spread. Points represent individual samples and are colored by aridity; gray lines connect measurements from the same soil profile across depths. Brackets indicate pairwise comparisons among soil layers. *p < 0.05, **p < 0.01, and ***p < 0.001; ns, not significant. All depth profile soils were sampled from temperate Inner Mongolia and the Loess Plateau in China (China_temperate; *n* = 9). SIC, soil inorganic carbon; *f*_modern_, the ^14^C‐derived modern carbon fraction of SIC.

Across the nine depth profiles from Chinese drylands, ∆^14^C‐SIC decreased with depth, from −689.9‰ (sensitivity range: −692.1, −687.8) in topsoil to −872.5‰ (−874.0, −871.0) in midsoil and −912.1‰ (−913.4, −910.7) in subsoil (Figure 4a; Table S1). *f*_modern_ showed the same pattern, declining from 29.7% (29.5, 30.0) in topsoil to 14.2% (14.0, 14.4) in midsoil and 10.4% (10.2, 10.6) in subsoil (Figure 4b; Table S1). If we assume that modern SIC has the ∆^14^C signature of atmospheric CO_2_ in the sampling year, the alternative estimate of *f*_modern_ (*f*_modern_′) changed only slightly (1.0–1.7 percentage points, Table S1). The ∆^14^C of respired CO_2_ was much higher than the ∆^14^C of SIC and also declined with depth (Figure 4a,d). Measurements of ∆^14^C‐SOC were available for fewer midsoil and subsoil samples because several low carbon samples could not be analyzed (Figure S3; topsoil: *n* = 9; midsoil: *n* = 5; subsoil: *n* = 3). The *δ* ^13^C of SIC was higher in topsoil (−4.3‰) than in midsoil (−7.2‰) and subsoil (−6.5‰) (Figure 4e). Along the same depth gradient, SIC content was lower in topsoil than in midsoil and subsoil, but similar in midsoil and subsoil (Figure 4c). Soil pH also increased significantly with depth (Figure 4f).

## 4 Discussion

**FIGURE 5:**
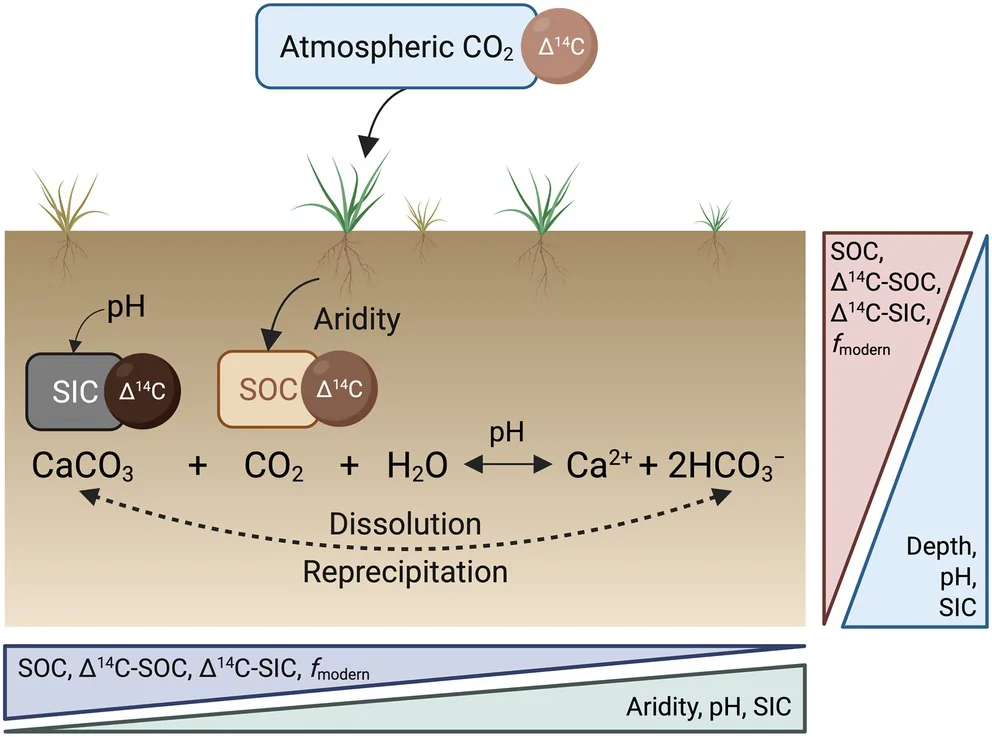
Conceptual framework linking the radiocarbon signatures of SOC and SIC in dryland soils. Pore‐space CO_2_ (derived mainly from root and rhizosphere respiration and SOC decomposition) dissolves in soil water and promotes carbonate dissolution. Subsequent carbonate reprecipitation allows the radiocarbon signature of soil CO_2_, which is linked to Δ^14^C‐SOC, to be imprinted on SIC. Lighter Δ^14^C circles indicate higher values and darker circles indicate lower values. With increasing aridity, reduced modern carbon inputs and older SOC lower the Δ^14^C of pore‐space CO_2_, resulting in lower Δ^14^C‐SIC. With increasing soil depth, SOC, Δ^14^C‐SOC, Δ^14^C‐SIC, and *f*_modern_ decrease, whereas pH and SIC generally increase. SOC, soil organic carbon; SIC, soil inorganic carbon; ∆^14^C‐SOC, ∆^14^C of SOC; ∆^14^C‐SIC, ∆^14^C of SIC; *f*_modern_, the ^14^C‐derived modern carbon fraction of SIC.

Our results provide radiocarbon evidence that topsoil SIC in drylands is not simply an inert reservoir of ancient carbonates. Across 42 Eurasian dryland sites, ∆^14^C‐SIC values were consistently far above ^14^C‐dead values, indicating that bulk SIC carries a measurable modern carbon imprint. This imprint was strongest in topsoils and weakened with increasing aridity and depth. A key result is that soil pH and ∆^14^C‐SOC, both structured by aridity, explained variation in ∆^14^C‐SIC better than climate or productivity alone. This pattern is consistent with a mechanism in which alkaline conditions limit carbonate dissolution and isotopic exchange, whereas younger SOC provides a modern carbon source for soil CO_2_ and thus for exchange with carbonate carbon (Figure 5). In the Chinese profiles, SIC in subsoils approached ^14^C‐dead values, indicating that deeper SIC stocks are dominated by inherited or ^14^C‐depleted carbonate carbon. Together, our results suggest that dryland SIC should not be treated as a uniform pool: topsoil SIC appears more closely coupled to modern carbon sources through pH‐mediated carbonate dissolution–reprecipitation, whereas subsoil SIC is more ^14^C‐depleted and more isolated from modern carbon sources.

Consistent with our hypotheses, ∆^14^C‐SIC and *f*_modern_ declined with increasing aridity across the Qinghai–Tibetan Plateau, as well as across the temperate drylands of China (after excluding two high‐leverage sites; Figure 1 and Figure S2). These patterns suggest that increasing aridity is associated with weaker modern carbon exchange with SIC, partly through higher soil pH and lower modern carbon inputs (Figure 3). Higher soil pH reflects more alkaline conditions that limit carbonate dissolution and isotopic exchange with modern carbon (Ferdush and Paul 2021; Zamanian et al. 2016). This interpretation is consistent with recent regional and global assessments showing that SIC stocks are vulnerable to soil acidification (Huang et al. 2024; Song et al. 2022; Tao et al. 2022). Lower pH is often associated with higher plant carbon inputs (Liu et al. 2022), and the positive correlation between ∆^14^C‐SOC and ∆^14^C‐SIC suggests that SOC acts as an important source of modern carbon for SIC. These results are consistent with the possibility that vegetation restoration in drylands may promote modern carbon exchange with SIC by increasing plant‐derived carbon inputs to SOC and lowering soil pH (Gong et al. 2017; Huang et al. 2022; Wang et al. 2011).

Our results suggest that the relationships of soil pH and Δ^14^C‐SOC with Δ^14^C‐SIC are not solely driven by aridity. When all available sites were considered, including the two high‐aridity, low‐pH sites in the Chinese temperate drylands, the relationship between aridity and Δ^14^C‐SIC weakened and was no longer significant, whereas soil pH and Δ^14^C‐SOC remained significant predictors of Δ^14^C‐SIC in the multiple regression analysis (Figure 2a). Previous studies have demonstrated substantial variation in carbonate composition and mineralogy among the regions represented in our SEM. For example, within the Chinese temperate drylands, carbonate mineralogy differs between the Chinese Loess Plateau, where calcite predominates (Li et al. 2013), and Inner Mongolia, where both calcite and dolomite occur (Zhou et al. 2025). Such differences may contribute to variation in soil pH independently of aridity and explain why sites with similar aridity exhibit different Δ^14^C‐SIC values. Therefore, the relationship between soil pH and Δ^14^C‐SIC should not be interpreted simply as a consequence of increasing aridity, but rather as reflecting the role of soil pH in regulating modern carbon exchange with SIC under both climatic and geological influences (Slessarev et al. 2016).

Our results show that ∆^14^C‐SIC declines with soil depth, indicating increasing contributions of ^14^C‐depleted carbonate carbon and weaker modern carbon exchange with SIC in deeper soil layers (Figure 4). The decline in Δ^14^C‐SIC with depth is likely related to the increase in soil pH and the decrease in ∆^14^C‐SOC with depth (Figures 4f and S3), which favor the preservation of ^14^C‐depleted or inherited carbonates and limit the availability of modern carbon for exchange with carbonate carbon (Zamanian et al. 2016). The ∆^14^C values in midsoil and subsoil layers were close to ^14^C‐dead, suggesting that downward transfer of modern dissolved inorganic carbon (DIC) had little influence on deeper SIC pools. The nearly ^14^C‐dead values in midsoil and subsoil layers may also partly result from upward transport of pre‐existing, ^14^C‐depleted carbonate, either in colloidal form or through dissolution and reprecipitation, without equilibration with the modern ^14^C of DIC. Such upward mobility means that we cannot rule out the possibility that ^14^C‐depleted SIC observed in deeper soils also undergoes dissolution, transport, and reprecipitation. Together, our results highlight the importance of soil pH and SOC inputs in shaping the depth distribution of SIC radiocarbon signatures and suggest that deeper SIC stocks are less influenced by modern carbon inputs.

Changes in ∆^14^C‐SIC were not associated with changes in SIC content across sites and depths, indicating that the amount of SIC does not necessarily reflect its age or degree of interaction with modern carbon. This extends recent stock‐based assessments of SIC vulnerability (Huang et al. 2024; Liao et al. 2025; Zeng et al. 2023) by showing that environmentally relevant changes in SIC can occur through isotopic exchange between ^14^C‐depleted carbonate carbon and modern carbon, even when SIC content remains unchanged. This decoupling between SIC content and radiocarbon signature is especially relevant in ecosystems where vegetation restoration leads to no significant change or even an increase in SIC stocks (Han et al. 2018; Zhao et al. 2024), while carbonate pools interacting with modern carbon sources would be sensitive to environmental changes such as increased acidity (Gocke and Kuzyakov 2011; Gocke et al. 2010). Recent work has further shown that routine soil processing typically removes coarse carbonate fragments (Batool et al. 2025), potentially leading to underestimation of SIC stocks, particularly in deeper soil layers. These coarse carbonate fractions are likely to contain older carbonate than the fine‐earth fraction, so both the size and the radiocarbon age of the deepsoil SIC pool may be greater than estimated from the fine‐earth fraction alone. The increase in SIC content from topsoil to midsoil and subsoil layers suggests that the modern carbon signal observed in topsoils likely reflects carbonate exchange or recrystallization, rather than net accumulation of modern carbonates to increase SIC content measurably in topsoils.

The depth‐dependent shifts in SIC sources also complicate the interpretation of *δ* ^13^C‐SIC. Although *δ* ^13^C‐SIC has been used to infer vegetation composition in paleosols (Cerling 1984), our results show that this proxy is not straightforward, especially in topsoils. *δ* ^13^C‐SIC values were lower in midsoil and subsoil than in topsoil (Figure 4e), and the estimated *δ* ^13^C of pore‐space CO_2_, derived from incubation *δ* ^13^C‐CO_2_ with a diffusive correction, approached isotopic equilibrium with SIC at depth but not in topsoils (Figure S4). The ^13^C enrichment of topsoil SIC relative to incubation‐derived CO_2_ can reflect a combination of several factors: atmospheric mixing (Hoefs 2009), C_4_‐derived inputs (Zamanian et al. 2016), inherited geogenic or dust‐borne carbonates (Kraimer and Monger 2009; West et al. 1988), and evaporative or degassing effects (Cerling 1984). Previous evidence from the Chinese Loess Plateau also shows aridity‐driven decoupling between pedogenic carbonate and soil organic matter *δ* ^13^C (Da et al. 2020), and further suggests that pedogenic carbonate *δ* ^13^C should be interpreted cautiously in paleoecological and Quaternary studies.

Although our radiocarbon analysis provides new insight into SIC age and sources, it has several limitations. First, radiocarbon measurements of bulk SIC integrate multiple carbonate pools that likely differ in age and origin, whereas our estimates of the modern carbon fraction in SIC were derived from a mass‐balance approach using simplified and approximate endmembers. Second, our study does not allow us to distinguish between two important processes: (i) net formation of new pedogenic carbonates and (ii) isotopic exchange or recrystallization of ^14^C‐depleted carbonates with modern carbon without changes in SIC content. Accordingly, a more modern ∆^14^C signature in SIC should not be interpreted directly as evidence for net carbonate sequestration. Third, our soil radiocarbon depth profiles are based on only nine sites and should therefore be interpreted with caution. Studies spanning a broader range of dryland ecosystems are needed to evaluate the generality of these patterns. Future work combining radiocarbon analyses of specific SIC fractions and soil pore‐space CO_2_, analyses of parent material and carbonate mineralogy, and long‐term manipulative experiments would help address these limitations.

In summary, our results show that topsoil SIC in dryland soils is not simply an inert legacy pool of ancient carbonates, but commonly contains a measurable and environmentally structured modern carbon imprint that links it more closely to contemporary ecosystem processes. The consistent relationships of ∆^14^C‐SIC with aridity, soil pH, and ∆^14^C‐SOC indicate that the modern carbon fraction of SIC is shaped by the joint effects of climate, soil geochemistry, and biologically derived carbon inputs. These findings suggest that environmental changes in drylands, including shifts in vegetation cover, carbon inputs, and soil acidity, may alter SIC not only through changes in stock size, but also through changes in the age and sources of carbonate carbon. In contrast, the near ^14^C‐dead signatures of subsoil SIC at the Chinese sites indicate that deeper carbonate pools remain dominated by inherited or ^14^C‐depleted carbonate carbon and are far less coupled to modern carbon sources. Overall, our study shows that dryland SIC should not be viewed as a uniform carbon reservoir and highlights the need to account for the strong vertical and environmental heterogeneity of SIC age and origin when evaluating carbon persistence and vulnerability under ongoing climate and land‐use change.

## Author Contributions

**Hui Wang:** conceptualization, methodology, investigation, visualization, writing – original draft, writing – review and editing, formal analysis, validation. **Jianbei Huang:** conceptualization, methodology, visualization, funding acquisition, project administration, supervision, writing – original draft, writing – review and editing, validation. **Fernando T. Maestre:** methodology, investigation, writing – review and editing, validation. **Guang Zhao:** methodology, investigation, writing – review and editing. **Nan Lu:** methodology, investigation, funding acquisition, project administration, writing – review and editing. **Cong Wang:** methodology, investigation, writing – review and editing. **Weiliang Chen:** methodology, investigation, writing – review and editing. **De Shorn E. Bramble:** methodology, writing – review and editing. **Marion Schrumpf:** methodology, supervision, writing – review and editing. **Michaela A. Dippold:** supervision, writing – review and editing. **Yangjian Zhang:** funding acquisition, project administration, writing – review and editing. **Sönke Zaehle:** funding acquisition, project administration, supervision, writing – review and editing. **Bojie Fu:** funding acquisition, project administration, supervision, writing – review and editing. **Susan Trumbore:** conceptualization, methodology, visualization, funding acquisition, project administration, supervision, writing – original draft, writing – review and editing, validation.

## Funding

This work was supported by Max‐Planck‐Gesellschaft, M.FE.A.EBIO0002; Chinese Academy of Sciences, HZXM20225001; and European Research Council, 647038 [BIODESERT].

## Conflicts of Interest

The authors declare no conflicts of interest.

## Supporting information


**Figure S1:** Locations of 42 dryland sites across Asia and Europe.
**Figure S2:** Relationships of ∆^14^C‐SIC (a) and *f*_modern_ (b) with aridity after excluding two high‐leverage observations in Chinese temperate drylands.
**Figure S3:** Variation in ∆^14^C‐SOC across soil layers.
**Figure S4:** Relationship between the δ^13^C of soil pore‐space CO_2_ derived from incubation and the δ^13^C of CO_2_ inferred to be in isotopic equilibrium with SIC.
**Table S1:** The ^14^C‐derived modern carbon fraction in SIC across soil layers.
**Table S2:** Multiple linear regression model of topsoil ∆^14^C‐SIC across drylands.
**Table S3:** Comparison of candidate piecewise structural equation models (SEMs) for the Chinese dryland dataset.
**Table S4:** The ^13^C‐derived modern carbon fraction in SIC across soil layers.

## Data Availability

The data that support this study are openly available in FigShare at https://doi.org/10.6084/m9.figshare.32034840.
