# Supplementary material for: Radiocarbon Reveals Modern Carbon Exchange With Topsoil Inorganic Carbon in Drylands
Source: Glob Chang Biol. 2026 Sep 4;32(9):e71095. doi: 10.1111/gcb.71095 (PMC13545485; doi:10.1111/gcb.71095)
Supplement: Supplementary file 1 [file GCB-32-e71095-s001.pdf]

Supplementary Information for

Radiocarbon reveals modern carbon exchange with topsoil inorganic carbon in drylands

Hui Wang, Jianbei Huang*, Fernando T. Maestre, Guang Zhao, Nan Lu, Cong Wang, Weiliang Chen, De Shorn E. Bramble, Marion Schrumpf, Michaela A. Dippold, Yangjian Zhang, Sönke Zaehle, Bojie Fu, Susan Trumbore

*Corresponding author: hjianbei@bgc-jena.mpg.de

Contents of this file

Figures S1–S4

Tables S1–S4

Supplementary Text

The ^{13}C-derived modern carbon fraction in SIC

To place the δ^{13}C values on a comparable basis, we assumed that carbonate was in equilibrium with CO_2 produced by SOM decomposition. We then calculated the δ^{13}C of CO_2 in soil pore air by correcting incubation-derived δ^{13}C-CO_2 by +4.4‰ for diffusive fractionation (Breecker et al., 2009; Cerling, 1984), with the resulting value hereafter referred to as δ^{13}C-CO_2-pore in equation (1). We also calculated the δ^{13}C of CO_2 expected to be in equilibrium with the observed SIC, assuming a calcite–CO_2 fractionation of ~9.6‰ at 20 °C (Romanek et al., 1992), with the resulting value hereafter referred to as δ^{13}C-CO_2-equilibrium in equation (2). In addition, we estimated the isotopic composition of plant-derived CO_2 from the observed C_3 and C_4 plant cover in the field surveys using mean δ^{13}C values of approximately −27‰ for C_3 plants and −13‰ for C_4 plants (Cerling et al., 1997), again including a +4.4‰ diffusive correction in equation (3).

$$\delta^{13}\text{C-CO}_2\text{-pore} = \delta^{13}\text{C-CO}_2 + 4.4 \quad (1)$$

$$\delta^{13}\text{C-CO}_2\text{-equilibrium} = \delta^{13}\text{C-SIC} - 9.6 \quad (2)$$

$$\delta^{13}\text{C-CO}_2\text{-plant} = C_{\text{C3}} \times \delta^{13}\text{C}_{\text{C3}} + C_{\text{C4}} \times \delta^{13}\text{C}_{\text{C4}} + 4.4 \quad (3)$$

$$C_{\text{C3}} + C_{\text{C4}} = 1 \quad (4)$$

where δ^{13}C-CO_2 is the δ^{13}C of soil respired CO_2 from incubation, δ^{13}C-SIC is the δ^{13}C of bulk SIC, and C_{C3} and C_{C4} are the proportions of total plant cover contributed by C_3 and C_4 plants, respectively.

Similar to f_{modern}, we estimated the ^{13}C-derived modern carbon fraction in SIC (F_{modern}) using the following mass balance equation (5):

$$\delta^{13}\text{C-SIC} = F_{\text{modern}} \times \delta^{13}\text{C-SIC}_{\text{modern}} + (1 - F_{\text{modern}}) \times \delta^{13}\text{C-SIC}_{\text{depleted}} \quad (5)$$

where δ^{13}C-SIC represents δ^{13}C-CO_2-pore, $\delta^{13}\text{C-SIC}_{\text{modern}}$ represents δ^{13}C-CO_2-plant, and $\delta^{13}\text{C-SIC}_{\text{depleted}}$ refers to δ^{13}C-CO_2-equilibrium (i.e., reflecting only carbonate rather than organic sources). For the alternative calculation presented in Table S4, measured topsoil and subsoil δ^{13}C-SIC values were used as proxies for the modern and depleted endmembers, respectively.

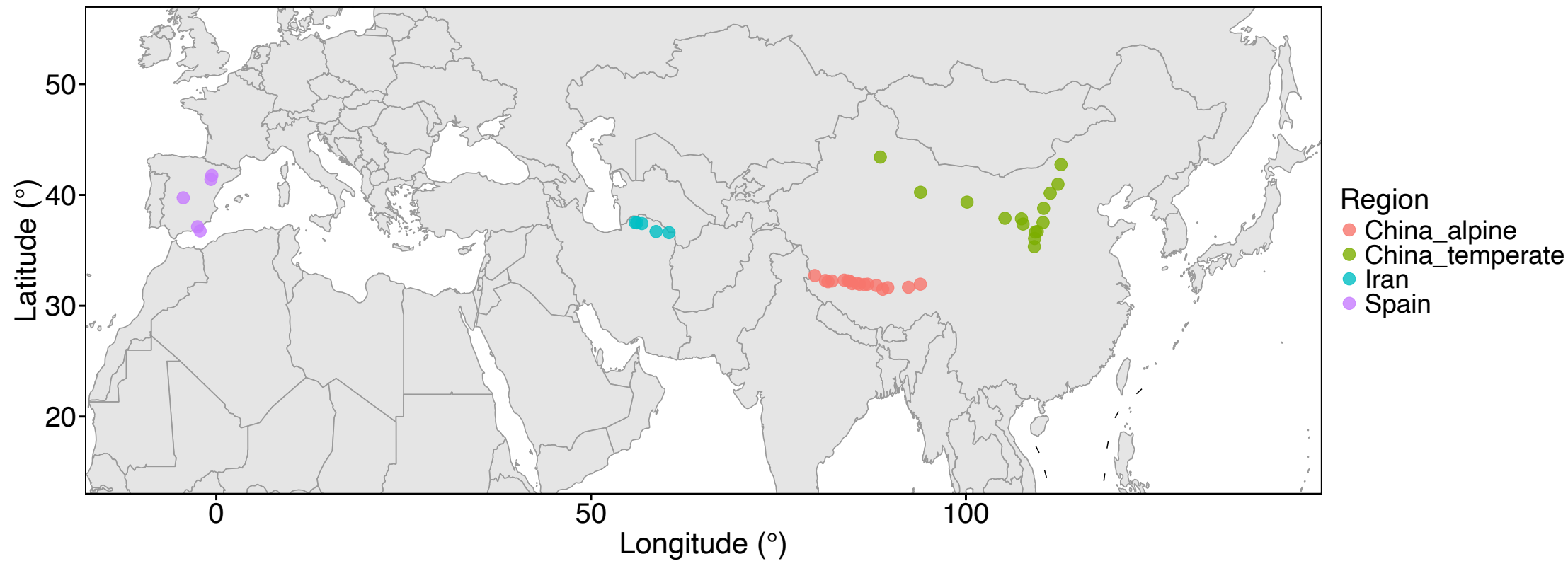

Figure S1. Locations of 42 dryland sites across Asia and Europe. Sites span four regions including China_alpine (*n* = 17), China_temperate (*n* = 15), Iran (*n* = 5), and Spain (*n* = 5). Map lines delineate study areas and do not necessarily depict accepted national boundaries.

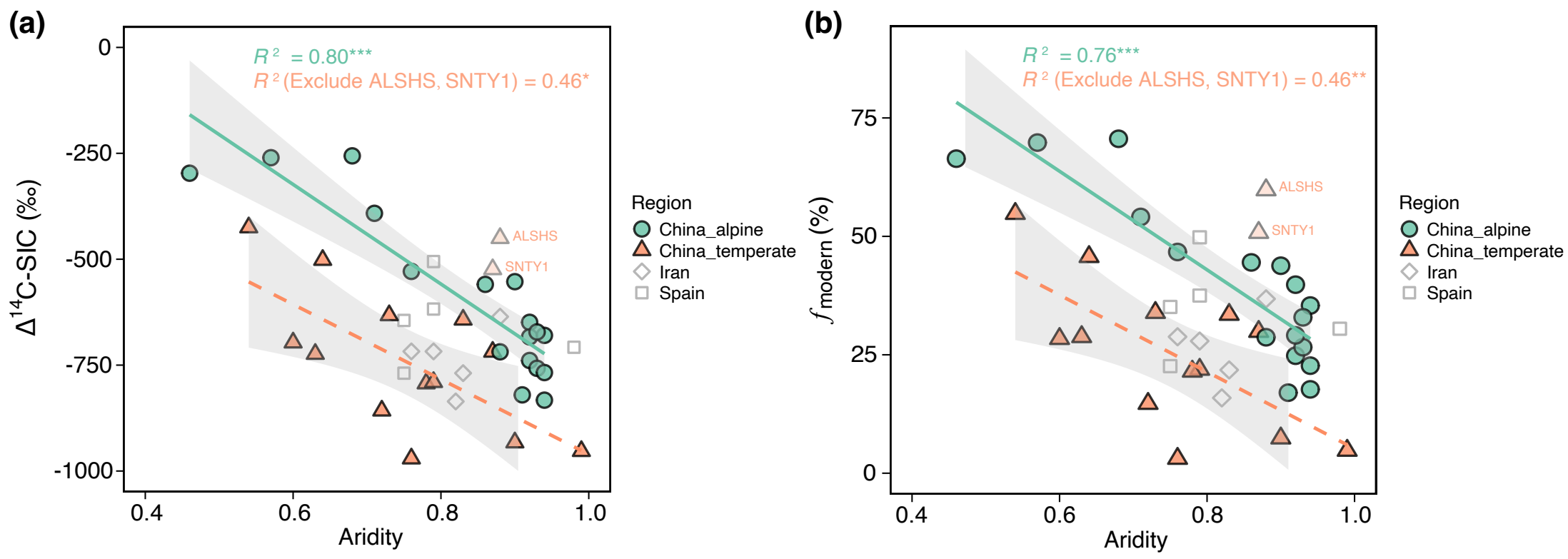

Figure S2. Relationships of Δ^{14}C-SIC (a) and f_{modern} (b) with aridity after excluding two high-leverage observations in Chinese temperate drylands. For the China_alpine samples, the green solid line shows the fitted linear regression, with gray shading indicating the 95% confidence interval (CI). For the China_temperate samples, ALSHS and SNTY1 are labeled as high-leverage observations, and the orange dashed line shows the fitted regression after excluding these two sites; gray shading indicates the 95% CI. The coefficients of determination (R^2) for the fitted relationships are shown in each panel. Significance levels are indicated as *$P < 0.05$, **$P < 0.01$, and ***$P < 0.001$. China_alpine, Qinghai–Tibetan Plateau in China; China_temperate, temperate Inner Mongolia and the Loess Plateau in China.

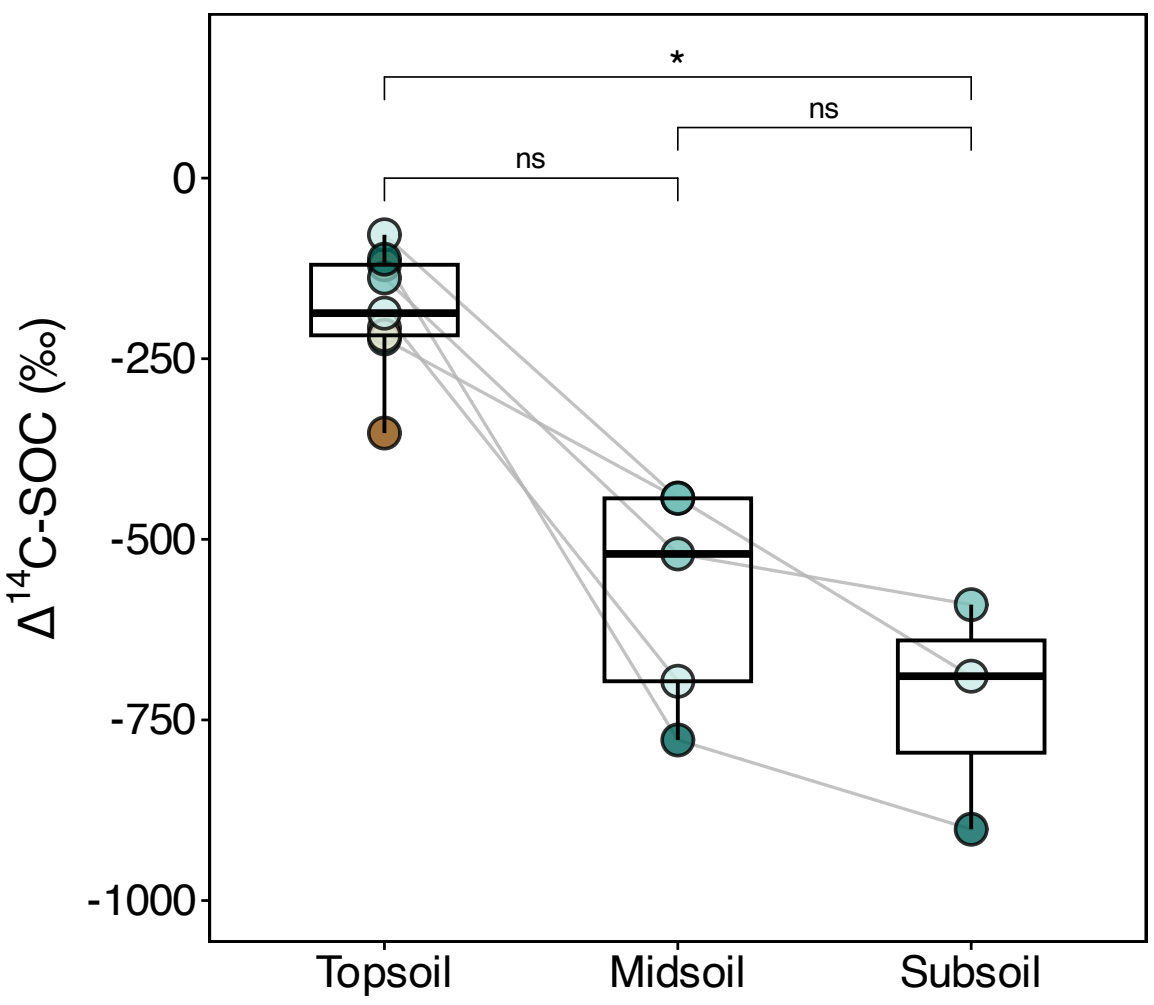

Figure S3. Variation in Δ^{14}C-SOC across soil layers. Boxplots show Δ^{14}C-SOC among topsoil (0–10 cm), midsoil (40–50 cm), and subsoil (90–100 cm), with the median and interquartile range indicated by the boxes and the data spread by the whiskers. Points represent individual samples and are colored by aridity. Brackets indicate pairwise comparisons among soil layers. *$P < 0.05$; ns, not significant. Sample sizes differed among soil layers because Δ^{14}C-SOC could not be measured in soils with low carbon content (< 0.1%). All depth-profile soils were sampled from temperate Inner Mongolia and the Loess Plateau in China (China_temperate; topsoil, $n = 9$; midsoil, $n = 5$; subsoil, $n = 3$).

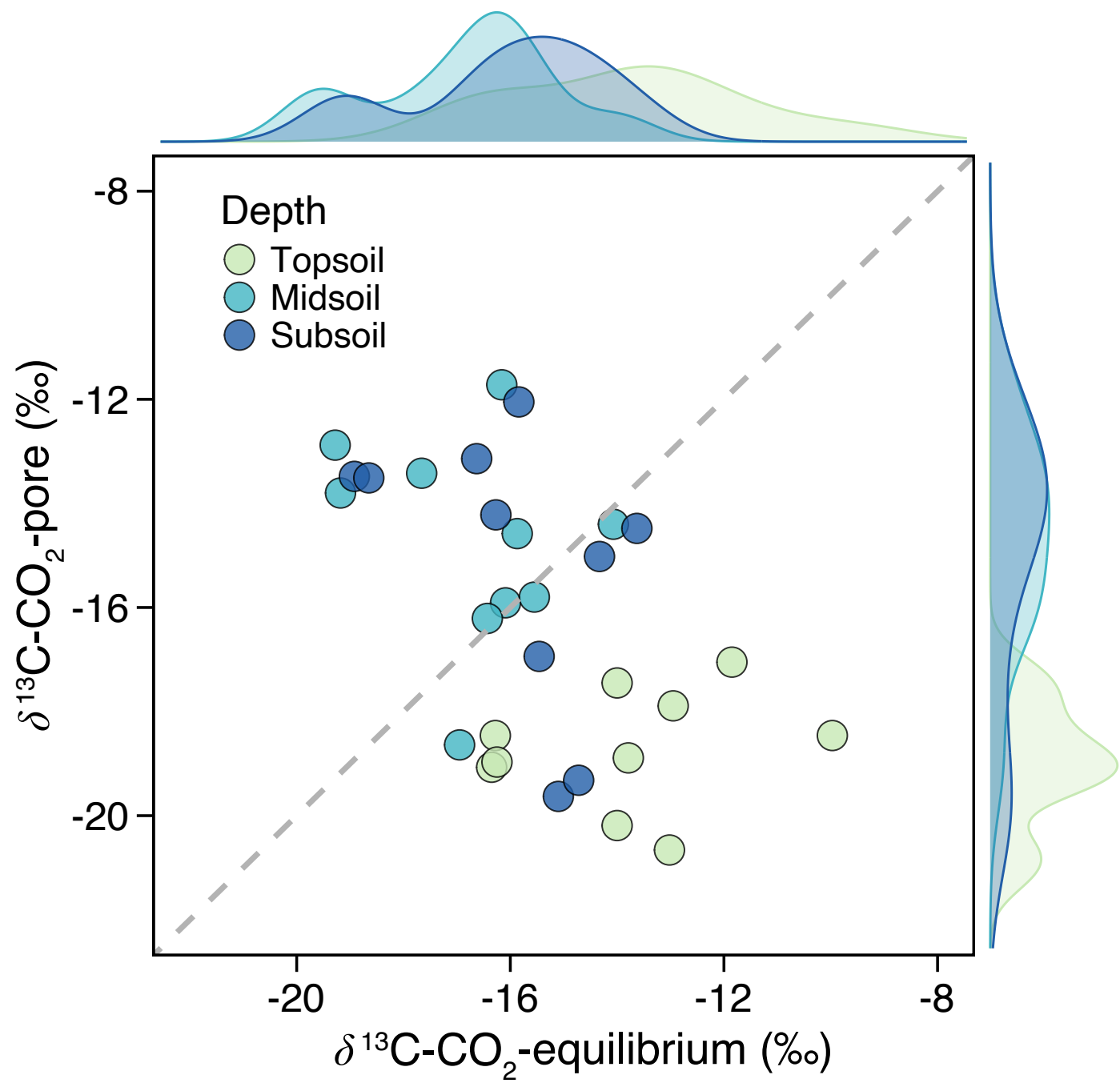

Figure S4. Relationship between the $\delta^{13}C$ of soil pore-space CO_2 derived from incubation and the $\delta^{13}C$ of CO_2 inferred to be in isotopic equilibrium with SIC. $\delta^{13}C$-CO_2-pore was calculated by adding 4.4‰ to the $\delta^{13}C$ of CO_2 respired during incubation to account for diffusive fractionation. $\delta^{13}C$-CO_2-equilibrium was calculated by subtracting 9.6‰ from $\delta^{13}C$-SIC, corresponding to the calcite–CO_2 fractionation at 20 °C. Data points in the scatter and density plots are colored by depth.

Table S1. The ^{14}C-derived modern carbon fraction in SIC across soil layers. f_{modern}, the ^{14}C-derived modern carbon fraction in SIC calculated by mass balance using the Δ^{14}C of soil respired CO_2 as Δ^{14}C-SIC$_{modern}$; f_{modern}', the ^{14}C-derived modern carbon fraction in SIC calculated by mass balance using the atmospheric Δ^{14}C signature as Δ^{14}C-SIC$_{modern}$' (n = 9). Δ^{14}C-SIC values are presented as mean ± error, with error indicating the systematic uncertainty. Averaged Δ^{14}C-SIC, f_{modern}, and f_{modern}' values are presented as mean [lower, upper], where the values in brackets represent the range obtained from the sensitivity analysis.

	**Topsoil**	**Midsoil**	**Subsoil**
**Δ^{14}C-SIC (‰)**	-632.3 ± 1.8	-856.0 ± 1.2	-905.1 ± 2.0
	-790.1 ± 2.5	-817.3 ± 0.9	-904.4 ± 0.7
	-874.8 ± 1.9	-922.2 ± 1.9	-935.9 ± 1.3
	-788.8 ± 2.8	-812.3 ± 1.2	-888.4 ± 1.2
	-857.4 ± 2.0	-902.3 ± 0.9	-948.7 ± 1.3
	-642.7 ± 2.0	-947.9 ± 1.9	-956.3 ± 1.4
	-501.8 ± 1.9	-895.7 ± 1.8	-926.7 ± 2.0
	-696.7 ± 1.8	-796.7 ± 1.8	-821.0 ± 1.8
	-424.9 ± 2.7	-901.9 ± 1.9	-922.1 ± 0.7
**Average**	-689.9 [-692.1, -687.8]	-872.5 [-874.0, -871.0]	-912.1 [-913.4, -910.7]
**f_{modern} (%)**	33.9	16.5	11.5
	21.9	19.4	10.9
	12.6	8.8	7.4
	22.1	20.6	13.2
	14.7	11.0	6.0
	33.5	6.6	5.2
	45.7	11.5	8.9
	28.4	22.1	21.2
	54.8	11.2	9.3
**Average**	29.7 [29.5, 30.0]	14.2 [14.0, 14.4]	10.4 [10.2, 10.6]
**f_{modern}' (%)**	36.9	14.4	9.5
	21.1	18.3	9.6

	12.5	7.8	6.4
	21.2	18.8	11.2
	14.0	9.6	5.0
	35.2	5.1	4.3
	49.1	10.3	7.2
	29.9	20.0	17.6
	56.6	9.7	7.7
**Average**	30.7<br>[29.5, 31.0]	12.7<br>[12.5, 12.9]	8.7<br>[8.6, 8.9]

Table S2. Multiple linear regression model of topsoil Δ^{14}C-SIC across drylands. The multiple linear regression model includes climate variables (mean annual temperature [MAT] and aridity [1 – aridity index]), a vegetation variable (net primary productivity [NPP], natural-log transformed), soil properties (pH, Clay + silt, cation exchange capacity [CEC], SIC, and SOC), and radiocarbon signatures (Δ^{14}C-SOC and Δ^{14}C-CO_2). Region was included as a categorical predictor to account for broad regional differences, with China_alpine used as the reference level. The model fit was R^2 = 66%, adjusted R^2 = 51%, $F_{13,28}$ = 4.24, P < 0.001, n = 42. Estimates are standardized coefficients; SE, standard error; CI, confidence interval.

Parameter	Estimate	SE	*t* value	*P* value	95% CI
**MAT**	0.06	0.12	0.54	0.60	[-0.18, 0.31]
**Aridity**	-0.09	0.23	-0.37	0.71	[-0.56, 0.39]
**NPP**	-0.15	0.19	-0.77	0.45	[-0.54, 0.25]
**pH**	-0.49	0.21	-2.33	**< 0.05**	[-0.92, -0.06]
**Clay + silt**	0.13	0.18	0.71	0.48	[-0.24, 0.50]
**CEC**	0.04	0.19	0.22	0.82	[-0.35, 0.44]
**SIC**	-0.23	0.18	-1.33	0.19	[-0.59, 0.13]
**SOC**	-0.09	0.24	-0.40	0.69	[-0.58, 0.39]
**Δ^{14}C-SOC**	0.47	0.20	2.35	**< 0.05**	[0.06, 0.88]
**Δ^{14}C-CO_2**	0.14	0.16	0.85	0.40	[-0.20, 0.47]
**China_temperate**	-0.65	0.36	-1.82	0.08	[-1.39, 0.08]
**Iran**	-1.33	0.58	-2.30	**< 0.05**	[-2.52, -0.15]
**Spain**	-1.59	0.68	-2.34	**< 0.05**	[-2.99, -0.20]

Table S3. Comparison of candidate piecewise structural equation models (SEMs) for the Chinese dryland dataset. Model fit was evaluated using Fisher's C statistic and AIC. The final model including aridity, pH, SOC, SIC, $\Delta^{14}C$-SOC, and $\Delta^{14}C$-SIC had the lowest AIC and an acceptable global fit, and was therefore retained. df, degrees of freedom; AIC, Akaike information criterion.

Model	Fisher's C	df	*P*	AIC
**Final model**	9.288	6	0.158	592.531
**Final model + texture**	26.533	14	0.022	594.421
**Final model + NPP**	21.205	14	0.097	706.247
**Final model + $\Delta^{14}C$-CO_2**	28.981	10	0.001	717.735

Table S4. The ^{13}C-derived modern carbon fraction in SIC across soil layers. For the calculation shown here, the topsoil and subsoil $\delta^{13}C$-SIC values were used as proxies for $\delta^{13}C\text{-SIC}_{modern}$ and $\delta^{13}C\text{-SIC}_{depleted}$, respectively, in equation (5). f_{modern}, the ^{14}C-derived modern carbon fraction in SIC; F_{modern}, the ^{13}C-derived modern carbon fraction in SIC ($n = 9$). Values are not shown (NA) where this method yielded unreliable F_{modern} estimates (i.e., >100% or negative).

$\delta^{13}C\text{-SIC}_{modern}$ (‰)	$\delta^{13}C\text{-SIC}_{depleted}$ (‰)	F_{modern} (by $\delta^{13}C$)	f_{modern} (by $\Delta^{14}C$)
-4.4	-4.7	40.1%	33.9%
-4.4	-5.1	NA	21.9%
-3.4	-6.7	79.7%	12.6%
-6.8	-9.3	43.5%	22.1%
-2.2	-5.9	NA	14.7%
-4.2	-4.0	NA	33.5%
-6.7	-6.2	NA	45.7%
-0.4	-7.0	NA	28.4%
-6.7	-9.1	NA	54.8%

References

Breecker, D. O., Sharp, Z. D., & McFadden, L. D. (2009). Seasonal bias in the formation and stable isotopic composition of pedogenic carbonate in modern soils from central New Mexico, USA. *Geological Society of America Bulletin, 121*(3–4), 630–640. https://doi.org/10.1130/B26413.1

Cerling, T. E. (1984). The stable isotopic composition of modern soil carbonate and its relationship to climate. *Earth and Planetary Science Letters, 71*(2), 229–240. https://doi.org/10.1016/0012-821X(84)90089-X

Cerling, T. E., Harris, J. M., MacFadden, B. J., Leakey, M. G., Quade, J., Eisenmann, V., & Ehleringer, J. R. (1997). Global vegetation change through the Miocene/Pliocene boundary. *Nature, 389*(6647), 153–158. https://doi.org/10.1038/38229

Romanek, C. S., Grossman, E. L., & Morse, J. W. (1992). Carbon isotopic fractionation in synthetic aragonite and calcite: Effects of temperature and precipitation rate. *Geochimica et Cosmochimica Acta, 56*(1), 419–430. https://doi.org/10.1016/0016-7037(92)90142-6
